# A national program to advance dementia research in Vietnam

**DOI:** 10.1186/s12913-024-10608-w

**Published:** 2024-02-01

**Authors:** Huyen Thi Thanh Vu, Tuan Anh Nguyen, Thu Thi Hoai Nguyen, Anh Trung Nguyen, Duyen Tran, Huong Nguyen, Ladson Hinton, Thang Pham

**Affiliations:** 1 Department of Geriatrics, Hanoi Medical University, 1 Ton That Tung Street, Dong Da District, Hanoi, Vietnam; 2 National Geriatric Hospital, Hanoi, Vietnam; 3 National Ageing Research Institute, Melbourne, Australia; 4 Department of Psychological Sciences, School of Health Sciences, Swinburne University of Technology, Melbourne, Australia; 5 UniSA Clinical and Health Sciences, University of South Australia, Adelaide, Australia; 6 Health Strategy and Policy Institute, Ministry of Health of Vietnam, Hanoi, Vietnam; 7 Department of Psychiatry and Behavioral Sciences, University of California, Davis, Sacramento, CA USA; 8 School of Nursing, University of Minnesota, Minneapolis, MN USA

**Keywords:** Research capacity building, Alzheimer’s disease, Dementia, Vietnam

## Abstract

**Background:**

As Vietnam and other low- and middle-income countries (LMIC) experience a rapid increase in the number of people living with dementia, an acute need exists to strengthen research capacity to inform policy, improve care and support, and develop national dementia plans. We describe the development and early outcomes of an National Institutes of Health (NIH)/National Institute on Aging (NIA)-funded national dementia research capacity building program in Vietnam.

**Methods:**

The research capacity building program commenced in 2019 and has three components: (1) Vietnam Alzheimer’s and other dementias research Network (VAN), (2) a mentored pilot grant program, and (3) research training, networking, and dissemination activities. The pilot grant program funds Vietnamese researchers for one to two years to conduct research focusing on Alzheimer’s Disease and Alzheimer’s Disease Related Dementias (AD/ADRD). Grants are reviewed and scored using NIH criteria, and priority is given to pilot grants with policy relevance and potential for future funding. An international pool of high-income country (e.g., United States, Australia, and United Kingdom) mentors has been engaged and mentors paired with each funded project. Training and networking activities include workshops on AD/ADRD research topics and regular meetings in conjunction with Vietnam’s annual national dementia/geriatric conferences. Dissemination is facilitated through targeted outreach and the creation of a national network of institutions.

**Results:**

Over four years (2019–2023), we received 62 applications, reviewed 58 applications, and funded 21 projects (4–5 per year). Funded investigators were from diverse disciplines and institutions across Vietnam with projects on a range of topics, including biomarkers, prevention, diagnosis, neuropsychological assessment, family caregiver support, dementia education, and clinical trials. A network of 12 leading academic and research institutions nationwide has been created to facilitate dissemination. Six research training workshops have been organized and included presentations from international speakers. Grantees have published or presented their studies at both national and international levels. The mentoring program has helped grantees to build their research skills and expand their research network.

**Conclusion:**

This research capacity building program is the first of its kind in Vietnam and may serve as a useful model for other LMIC.

**Supplementary Information:**

The online version contains supplementary material available at 10.1186/s12913-024-10608-w.

## Introduction

Low- and middle-income countries (LMIC) are undergoing a demographic transition that will result in a dramatic increase in the number of older people, including those living with dementia. By 2030, LMIC will be a home for about 70% of people living with dementia globally [1]. As a LMIC, Vietnam is experiencing a similar trend with the number of people living with dementia estimated to double every 20 years, from 660,000 in 2015 to 1.2 million by 2030 and 2.4 million by 2050 [2, 3].

Dementia is a major public health challenge in LMIC, being among the most disabling and costly diseases to afflict older adults [4]. The majority of people living with dementia are cared for in the home by family members who often sacrifice time and income to provide care. Changed behaviors, such as agitation and anxiety, are particularly stressful for caregivers, who themselves are at increased risk of adverse mental and health outcomes, as well as institutionalization [5]. Strengthening dementia research capacity is recognized as a central part of national dementia plans to generate evidence for policymakers to develop effective dementia prevention, diagnosis, treatment and care, and support for people living with dementia and their family caregivers.

Vietnam faces a gap in dementia research capacity to generate evidence to guide policymakers [2]. This creates an acute “evidence gap” that threatens progress to effective policy development. These dual challenges—lacking scientific evidence to guide policymakers and limited capacity to generate this evidence—were highlighted at the first Vietnam National Dementia Conference held in Hanoi in September 2018 [2]. There is now growing recognition in the Ministry of Health and the scientific community that building dementia research capacity in Vietnam is an important priority.

Strengthening research capacity in LMIC is recognized as a critical yet often challenging issue for the advancement of global health [6]. While many barriers to capacity building exist, many programs have a central focus on training, network-building among scientists within a given country and regionally, and on the importance of promoting leadership in research among LMIC scientists [7, 8]. In addition, capacity building needs an explicit focus of research development (in addition to the research component) to foster mutually beneficial and equitable collaborations between investigators in LMIC and high-income countries (HIC), and attention to research ethics [8–12].

Based on these principles, this paper describes the design, implementation, and early results of a capacity building program to strengthen dementia research in Vietnam. Dementia research was defined very broadly to include a range of topics including prevention, early diagnosis, family caregiving, support of family caregivers, and end-of-life care for people living with dementia. Lessons learned to date from this international collaboration are also described. To our knowledge, this is among the first research capacity building programs focusing specifically on building the workforce to conduct dementia-related research in Vietnam.

## Methods

### Overview

This program is part of an National Institutes of Health (NIH) funded grant (R01 AG064688) that includes both a research capacity building component and a clinical trial to test the efficacy of a family dementia caregiver intervention in Vietnam [13]. Our dementia research capacity building program in Vietnam includes several approaches to capacity building, including the development of a formal research network to facilitate collaboration among investigators within Vietnam, “hands-on” learning through mentored pilot projects, placing National Geriatric Hospital (NGH) investigators in leadership roles, and mini-workshops offered by international experts [14]. Our proposed approach to capacity building also draws on a mentored pilot grant program that has been used successfully by NIH funded centers, including the Resource Centers for Minority Aging Research (RCMAR) [15]. We now describe each aspect of the research capacity building program, as well as the results of this program.

### Vietnam Alzheimer’s and other dementias research Network (VAN)

In collaboration with a newly formed national organization—the Vietnam Association of Geriatrics and Gerontology (VAGG), we established VAN as a national network of institutions supporting dementia research. VAN was developed to serve as a platform to support the Mentored Pilot Grant Program and is composed of 12 research institutions and groups across Vietnam (see Table 1).

**Table 1:** Institutions in the VAN network

No	Institution/Group	Institution type
1.	National Geriatric Hospital	Clinical
2.	Bach Mai Hospital	Clinical
3.	Hanoi Medical University	Academic
4.	Vietnam National University	Academic
5.	Hanoi University of Pharmacy	Academic
6.	Hanoi University of Public Health	Academic
7.	Hanoi National University of Education	Academic
8.	Hanoi Medical College	Academic
9.	University of Medicine and Pharmacy at Ho Chi Minh City	Academic
10.	Dinh Tien Hoang Institute of Medicine	NGO
11.	Institute of Population, Health and Development	NGO
12.	Health Strategy and Policy Institute	Government

The network includes interdisciplinary researchers from clinical and academic institutions, non-governmental organizations (NGO), and government agency for policy making. The network started with institutions and groups from the North and South of Vietnam. In each institution/group, leaders and key individuals such as head of scientific research department help to identify and introduce researchers who are interested in conducting dementia-related research.

### VAN mentored pilot grant program

The overall objective of the pilot grant program is to attract and support talented Vietnamese researchers (both established scientists and early career researchers) who wish to conduct Alzheimer’s Disease and Alzheimer’s Disease Related Dementias (AD/ADRD) research, including prevention, early diagnosis and assessment, care and services for people living with dementia, and support for family caregivers. The program seeks to identify both researchers with experience in conducting dementia research as well as those who are working in other areas but wish to move into the dementia field.

The pilot grant program includes a pre-application phase for outreach and cultivation of potential applicants, activities related to application review and selection, and support of pilot grant awardees. The steering committee of the pilot grant program provides outreach and support prior to application submission, serves as a point of contact for prospective applicants, works with applicants to facilitate pre-submission review and consultation, and takes the lead in organizing review of pilot applications and identifying reviewers.

The pilot grant program is open to Vietnamese faculty at all levels, from post-graduate students to full professors. No prior experience in conducting dementia research is required, but applicants are expected to have research experience and skills to conduct their project independently. An application template (see**Appendix**) is provided, and applications need to be prepared in both Vietnamese and English. Maximum of five pages is allowed for the proposal, including the following sections: (1) title, (2) aims and hypotheses, (3) background/review of literature, (4) research methods, (5) data analysis plan and power calculation, (6) timeline for study completion, and (7) risks and benefits. Each application also includes a brief statement of the applicant’s qualifications and a plan to achieve outcomes (e.g., publications, future grant applications). A detailed budget, curriculum vitae, and letter(s) of support from their home institution or department are also required. The grant calls focus on four main priority areas, which were identified by national stakeholders at the launch of the VAN, nested in the second Vietnam National Dementia Conference in 2019. The four areas identified are: (1) dementia prevention and risk factors, (2) dementia detection, screening, and diagnosis, (3) living with dementia—providing supports for people living with dementia, and (4) providing supports for caregivers.

Applications are reviewed using a scoring template developed based on the NIH grant scoring system by a panel, including one research team member, one member with sufficient methodological expertise appropriate to the proposals (e.g., statistician and/or qualitative methodologist), and at least one external scientist. To support capacity building for early career researchers, lead applicants need to identify at least one early career researcher (e.g., graduate student, postdoctoral) as part of their research team. Criteria for review are novel and important dementia-related science with high potential for policy impact, technically sound methods, potential for follow-up studies, and likelihood of future funding. All applicants will need to commit to attend VAN quarterly networking meetings and present their pilot grant findings.

The pilot grant program funds four to five pilot awards each year with a maximum amount of US $10,000/award. The successful Vietnamese applicants are allowed to undertake their dementia research project within two years and the information about their project is available on our project’s website (https://sasuttritue.vn/).

Each grantee is matched with at least one HIC mentor, who also serves as collaborators (see Table 2). HIC mentors can be identified by grantees or the VAN steering committee. Pilot awardees work with their HIC mentor and the VAN steering committee to identify any additional training needs. Monthly mentoring meetings between HIC mentors and their respective mentees are organized using videoconference apart from frequent email contact as needed.

**Table 2:** List of mentor institutions and areas of expertise

Country, Institution	Expertise
**United States of America**	
University of California, Davis	Neuropsychology, Social Psychology, Geriatric Psychiatry, Biostatistics, Emergency Geriatrics
University of California, San Francisco	Health Policy & Management
Alliant International University	Clinical Psychology
Tulane University	Public Health
University of Minnesota	Social Work
**Australia**	
National Ageing Research Institute	Pharmacy, Public Health, Community Medicine, Digital Health, Cultural Diversity
University of Sydney	Geriatrics and Gerontology, Mental Health, Healthy Aging
University of New South Wales	Psychiatry, Aged Health Care, Geriatrics and Gerontology
Swinburne University of Technology	Psycho-Oncology Research, Behavior Change, Medication Adherence
University of Queensland	Health Economics
University of South Australia	Pharmaceutical Science
**United Kingdom**	
Newcastle University	Primary Care, Aging

All awardees complete online training in human subject research and obtain local Institutional Review Board (IRB) approval at institutions that have Federal-Wide Assurance Numbers. Prior to receiving pilot funds, grantees need to submit documentation of online course completion and local IRB approval. The IRB at University of California, Davis also provides oversight for pilot grants that involve human subjects research.

Upon completion of their project, grantees are asked to complete the final progress report. We also ask both mentors and grantees to complete mentoring evaluation surveys. The final progress report and evaluation templates (see **Appendix**) are developed and adapted based on the NIH templates for these activities.

Yearly survey and report to track outcomes: For each pilot awardee during and after completion of their study, we will track publications (Vietnamese and international journals), grant applications, grants funded, policy impact, and career advancement.

### Research training, networking, and dissemination activities

In the first year, there was a large-scale kick-off networking meeting (National Dementia Forum) to advertise the Pilot Grant Program whereas in the fifth year, another large-scale National Dementia Forum will be organized to disseminate the project results. Apart from these first and last meetings, quarterly online networking meetings with attendance of 30–40 participants have been organized with presentations from awardees and dementia experts. We invite experts from Vietnam and HIC to share their research experience or give lectures on different dementia research topics. Every quarter, the VAN network sends an invitation and agenda to grantees and interested individuals to register via Google Form. We open research training meetings to all grantees, prospective applicants, and interested researchers. These networking meetings have several purposes, including promoting collaboration among different individuals and groups in Vietnam, research training, and providing an opportunity for investigators including early career researchers to share their work.

## Results

### Characteristics of applications reviewed and funded

**Fig. 1:**
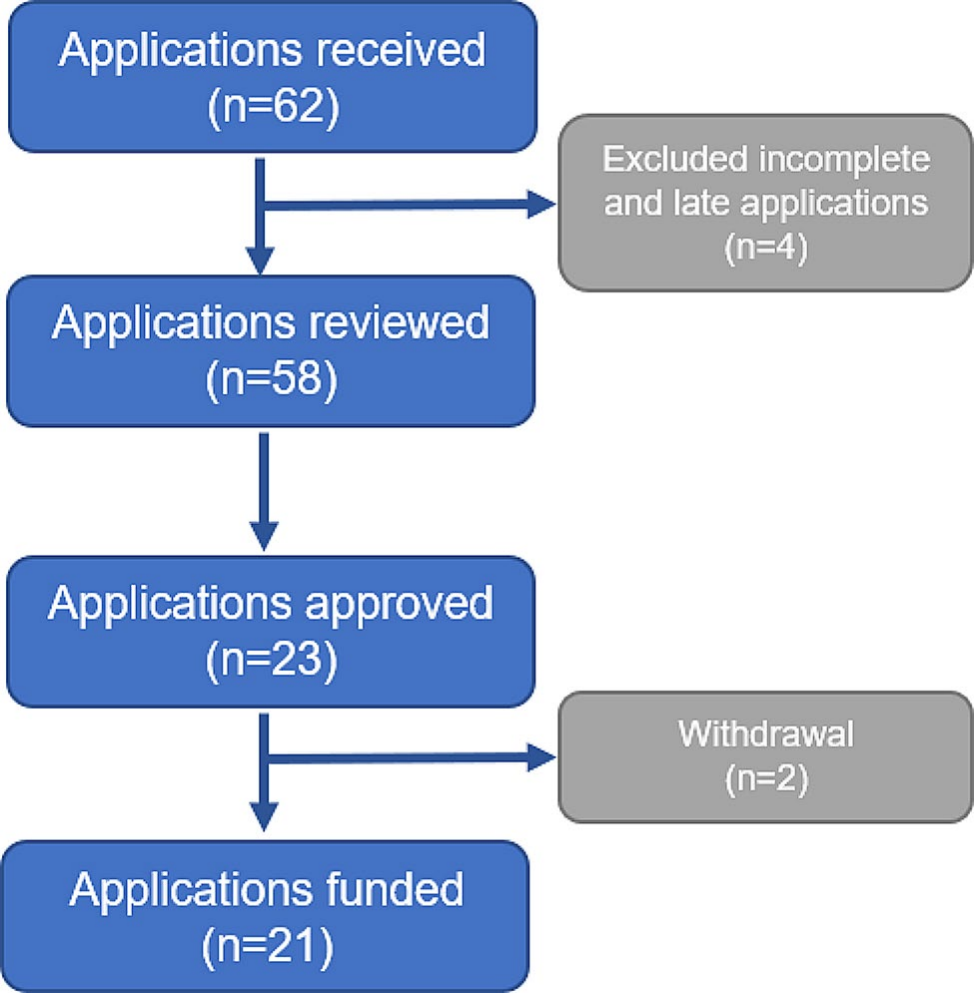
VAN pilot program results in four years (2019–2023)

Over four years (2019–2023), we received 62 applications, reviewed 58 applications, and funded 21 projects (see Fig. 1).

Most proposals were for quantitative research with 13 projects in year 1, 3 projects in year 2, 11 projects in year 3, and 8 projects in year 4. The applicants’ professional fields were diverse, including medicine (31 projects), public health (7 projects), nursing (5 projects), biomedical sciences (3 projects), social work (4 projects), psychology (2 projects), and pharmacy (6 projects).

In the first year, there were 27 submitted proposals, of which one late and one incomplete applications were excluded. After the review process, eight proposals were selected for funding. However, two projects were withdrawn due to the PI’s job change, leaving six projects being funded. There were four projects from North Vietnam and two projects from South Vietnam. The grantees’ professional fields were diverse, including medicine (3 projects), public health (1 project), nursing (1 project), and biomedical sciences (1 project).

In the second year, there were eight submitted proposals, of which five were funded. Three of them were from North Vietnam and the remainders were from South Vietnam. The grantees’ professional fields were medicine (4 projects) and public health (1 project).

In the third year, there were 14 submitted proposals, of which one late submission was excluded. In total of five selected projects, the grantees’ professional fields were diverse including medicine (2 projects), public health (1 project), nursing (1 project), and pharmacy (1 project).

In the fourth year, there were 13 submitted proposals, of which one late submission was excluded. The committee chose five proposals for funding. The grantees’ professional fields were medicine (2 projects), social work (2 projects), and pharmacy (1 project).

**Fig. 2:**
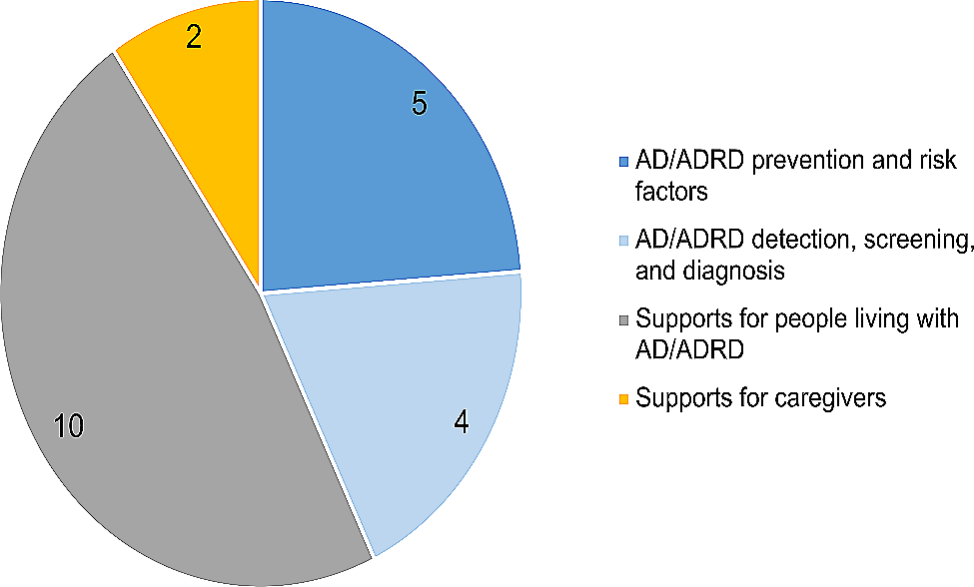
The number of funded projects by topic

The research focus of the 21 funded projects were spread across four identified dementia research priorities: five in dementia prevention and risk factors, four in dementia detection, screening, and diagnosis, ten in providing supports for people living with dementia, and two in providing supports for caregivers (see Fig. 2 and Appendix for the list of funded projects).

To date, five out of 21 funded projects have completed the study with an average duration of 2.4 years. Of the five completed projects, three projects were completed in two years and two projects were completed in three years.

### Publications, posters, and presentations from grantees

There have been outstanding results from the pilot funding program. To date, there have been five national peer-reviewed publications and four international peer-reviewed publications. There have been ten international presentations and nine national presentations. The grantees have disseminated results of their pilot studies at conferences or journals relating to geriatrics, brain health, and dementia. More publications, posters, and presentations are anticipated in the future since several studies are ongoing or in the write-up phase.

### Evaluation results in working with mentors

We conducted a short evaluation survey in working with mentors. We received 17 responses in total of 21 grantees. The results showed that 59% grantees have worked with their mentor more frequent than every two months. Grantees reported benefits from the mentoring program including being able to learn new knowledge and research skills from mentors and expand their network with experts in the field. However, there were some challenges faced by grantees. The time zone difference between mentors and grantees was one of the biggest challenges. Lack of responsiveness from some mentors was another challenge.

### Outcomes of research training, networking, and dissemination activities

There were six research training and networking webinars from 2020 to 2022. The main purpose of these webinars were for grantees to report their research progress, discuss the challenges in conducting research, as well as to receive research training through didactic presentations by HIC and Vietnamese experts. The first meeting was held in person at the National Geriatric Hospital. In this meeting, there were multiple topics including “Grant opportunities for Vietnamese researchers,” “The collaboration with international researchers,” “How to write a winning grant proposal: An expert guide,” “Developing social work and social science research about dementia and mental health among aging populations in Vietnam,” and “Learning experience from the first round: Funded and rejected proposals.” We sent an evaluation form to attendees after the meeting for their evaluation and for them to indicate their topic preference in the next webinar based on their needs and interests.

Due to the COVID-19, subsequent meetings were held virtually. The research training topic for the second meeting was “Pilot intervention research: Design and methodological issues,” and the third meeting was “How to write a good and quick paper” and “Perspective of editors in submitting international publications.” To support grantees to deal with COVID-19 situations, the topic for the fourth meeting was “Conducting research during the COVID-19 pandemic.” In the fifth webinar, we invited speakers from Japan to give a talk entitled “Overview about dementia in Japan, Community-based dementia interventions: a) green care farms for the people with dementia, and b) temple-based cafes for the family caregivers” and “Research studies on institutional care staff conducted through collaboration between Buddhist priests and researchers.”

In the sixth webinar, researchers from the two completed projects presented their study results and shared their experiences in conducting their pilot studies. There were some shared experiences including the process of applying for IRB approval, working regularly with mentors and supports from VAN officers.

In the last four years since the commencement of the capacity building program, the VAN steering committee members have presented the initial results of our program at both international and national conferences/workshops. There was one poster presentation at the Alzheimer’s Association International Conference in 2022 and three oral presentations at the first, second, and third Vietnam National Geriatric Conference. Two funding announcements were launched at a Scientific Research workshop of the Department of Cardiology at Hanoi Medical University in 2022 and Hanoi Medical University Scientific Conference in 2022.

## Discussion

To our knowledge, this pilot funding program is the first of its kind to systematically strengthen dementia research capacity in Vietnam. We started with the first national stakeholder workshop nested in the first Vietnam National Dementia Conference to identify the top priorities in dementia research in Vietnam. After four years, we have established a research network with 12 institutions across Vietnam and funded 21 projects.

Apart from our current pilot program, there is a handful of dementia research capacity building initiatives in Vietnam, including those in our NHMRC-NAFOSTED project (APP1154644) to develop Vietnam’s National Dementia Plan [2] and NHMRC-eASIA project (APP2001548) to develop and test an iSupport Virtual Assistant to empower dementia caregivers in Australia, New Zealand, Indonesia, and Vietnam [16]. In the former, the dementia research capacity was built using “hands-on” training on policy, epidemiological and qualitative analyses. In the latter, an additional dedicated component of four intensive training workshops on Theory of Change, understanding dementia through the lens of digital technology, development of digital health platform, and cultural adaptation of evidence-based carer interventions was in place.

A few dementia research capacity building programs are also in place in other LMICs. Leroi et al. reported two exemplars of their dementia research capacity building at individual level in India, Pakistan, and Bangladesh [17]. The UK-funded “Strengthening Response to Dementia in Developing Countries” project is another initiative that builds capacity for dementia research in seven LMICs [18]. Like our NHMRC-NAFOSTED and NHMRC-eASIA projects, these are “integrated capacity development” initiatives where the research capacity was built for internal local researchers, who were project team members, using training to conduct the research activities of the main research project [19]. Our current pilot program builds on these experiences, expanding its dedicated capacity building component to fund pilot projects for external local researchers, who brought their own dementia research topics under the scope of dementia research priorities identified by Vietnamese national dementia stakeholders. In addition, our pilot program has also brought in external HIC mentors to support pilot awardees, thus broadening the international collaboration for Vietnamese dementia researchers.

There are several lessons learned through our dementia research capacity building initiative in Vietnam. First, Vietnam has yet to establish a national dementia registry. While at the institutional level, some healthcare facilities such as National Geriatric Hospital have data on their patients with dementia in medical records, the quality of medical records significantly varies across regions and levels of care [20]. In addition, the unclear data dissemination mechanism in Vietnam will likely restrict data use in research. This creates challenges for dementia researchers to identify dementia cases without having specialists doing patient screening.

Second, most applications to our pilot funding program are particularly weak in terms of methods, despite their relatively significant research topics. Key reasons have been identified, including lack of grant writing skills, lack of understanding research methodology, and lack of relevant published literature in the dementia field in Vietnam. Moreover, many applicants were not familiar with ethics requirements for conducting human research leading to delays in obtaining ethics approval, both locally and from NIH. In response, we have developed training webinars open for both grantees and prospective pilot applicants, covering a variety of topics including grant writing, research methods, and cognitive assessments. The NIH model where applicants can seek feedback from a dedicated program officer during the proposal development process might be a good approach to help improve the proposal quality.

Third, the number of applications to our pilot funding program reduced significantly from 27 applications in the first year (2019) to 8 applications in the second (2020) and then increased to 14 applications in the third year (2021) and 13 applications in the fourth year (2022) respectively. Some potential explanations have been identified. The primary approach to promote our pilot program was through our annual National Dementia Conferences, which was organized in 2019 but was canceled in 2020 and 2021 due to COVID-19. Also, institutional visits by VAN steering committee members to potential research groups in the North, Central, and South of Vietnam were conducted in the first year, but not in the subsequent years also due to COVID-19. The former approach led to the limitation of the applicants and then grantees mainly being from medicine disciplines. The latter approach might be a supplement to target research groups from other disciplines such as educational, social, and cultural areas.

Our pilot program has several limitations. Equipping local researchers with the skills to translate their research findings into policy and practice is a fundamental aspect of a research capacity building program [17]. However, our program has focused on pilot studies that are modest in size but allow researchers to develop research skills and generate pilot data that can become the basis of larger and potentially more impactful research in the future. Another and related limitation is that our program did not have a major focus on grant writing skills to support investigators in building upon the results of their pilot studies to apply for extramural grant funding. 

Our pilot program is similar in some respects to a pilot project grant program in the US, run by Washington State University, which has funded three cohorts of a total of 13 grantees working in dementia and health disparities research [21]. These awardees received 18-month program, with the first 12 months conducting their approved pilot project and the last 6 months for a grant writing training. They had regular in person/Zoom meetings that include didactic sessions for every three to four months and bi-weekly interactions with their mentor team, which is similar to our program. The difference is that they had a five-day intensive grant-writing workshop in Month 13 and a subsequent dedicated six months for supporting awardees to develop a new grant proposal with a mock review of their funding proposal in Month 18. This might help the trainees to obtain external funding after their pilot project finishes. Our pilot program touched base on grant writing skills through a grant writing webinar, although it is less intensive. We will address our limitations by strengthening and having a dedicated grant writing component to support grantees to seek future funding from 2023. We will also include training in patient and public involvement (including awareness raising, stigma and health literacy) and advocacy for translating research findings into policy and practices.

### Future directions

We plan to use the experience and lessons learned from this research capacity building program to continue our efforts in the future. In particular, we plan to strengthen our program to assist Vietnamese investigators in grant-writing to enable them to garner extramural support and to establish their own programs in AD/ADRD research. In addition, future efforts will focus on further outreach to investigators and institutions in the central and southern parts of Vietnam. Because many investigators were not able to complete data collection within one year, we may also consider offering two-year pilot projects, particularly for those proposing primary data collection. Finally, the pilot grant review process may benefit from the inclusion of additional stakeholders, including older adults including those living with dementia and care partners.

## Conclusion

This paper provides information about the first comprehensive dementia research capacity building initiative in Vietnam and shows our achievement and lessons learned in the last four years of the initiative. It may serve as a model for other LMICs wanting to build their dementia research capacity in particular and their research capacity in general.

### Electronic supplementary material

Below is the link to the electronic supplementary material.

Supplementary Material 1

Supplementary Material 2

Supplementary Material 3

Supplementary Material 4

Supplementary Material 5

## Data Availability

No datasets were generated or analysed during the current study.
