# Supplementary material for: A national program to advance dementia research in Vietnam
Source: BMC Health Serv Res. 2024 Feb 1;24:156. doi: 10.1186/s12913-024-10608-w (PMC10832103; doi:10.1186/s12913-024-10608-w)
Supplement: Supplementary file 1 — Supplementary Material 1 [file 12913_2024_10608_MOESM1_ESM.doc]

**COVER PAGE**

Project title:

| Name of Principal Investigator (PI) |  |
| --- | --- |
| Position / role of PI | Position:  Role: |
| Address of PI: |  |
| Email address:  Telephone: |  |
| List of all people and the departments / institutions that will be involved with this project |  |

**RESEARCH PROPOSAL**

***(This research proposal should be a MAXIMUM of 5 pages in total, excluding the cover page and references. Font Arial size must be 12-14 point, CVs, detailed budget, and letter of support are required. Each of the elements listed below should be addressed in the application.)***

1. **Title**
2. ***Aims and hypotheses***
3. **Background/review of literature**
4. **Research methods**
5. **Data analysis plan and power calculation**
6. **Timeline for study completion** *(including realistic timeframe for project approvals)*
7. **Risk and benefits**
8. **References**
