# Supplementary material for: A national program to advance dementia research in Vietnam
Source: BMC Health Serv Res. 2024 Feb 1;24:156. doi: 10.1186/s12913-024-10608-w (PMC10832103; doi:10.1186/s12913-024-10608-w)
Supplement: Supplementary file 2 — Supplementary Material 2 [file 12913_2024_10608_MOESM2_ESM.docx]

VAN MENTORING PROGRAM

Dear Mentees,

Thank you for your participation in the pilot funding scheme in our NIH - R01 project “Advancing Alzheimer’s family caregiving intervention and research capacity in Vietnam”!

Please take a few moments to complete the survey questions below. Your responses will remain completely anonymous. Your feedback will help us improve our mentoring program to support Vietnamese investigators who conduct dementia research in Vietnam.

If you have any questions, please contact us at [vanprograms@gmail.com](mailto:vanprograms@gmail.com). Sincerely,

Vietnam Alzheimer’s and other dementias research Network (VAN) Steering Committee

Part 1: Survey

Directions: Click one selection per question which best describes your opinion regarding your mentor

1. Was your mentor easy to approach and talk with?

*Mark only one oval.*

Yes No Maybe

Don't know N/A

1. Did your mentor review and discuss with you your career/individual development plan or the two of you just focused on undertaking your funded pilot project?

*Mark only one oval.*

Career/individual development plan

Focus on undertaking the funded pilot project Both

1. Did your mentor offer advice and encouragement to you with respect to your goals and objectives?

*Mark only one oval.*

Yes No Maybe

Don't know N/A

1. Did the two of you meet regularly?

*Mark only one oval.*

Yes No Maybe

Don't know N/A

5a. If answer is Yes to question 4: How often did the two of you meet?

5b. If answer is No to question 4: Why was that?

6. Did you receive regular feedback?

*Mark only one oval.*

Yes No Maybe

Don't know N/A

7a. If answer is Yes to question 6: Was the feedback helpful to you? Why or why not?

7b. If answer is No to question 6: Why was that?

1. Did your mentor involve you in networking, or suggest professional organizations to help build competency in the objectives you identified?

*Mark only one oval.*

Yes No Maybe

Don't know N/A

1. Did your mentor solicit your thoughts and opinions when making suggestions or recommendations?

*Mark only one oval.*

Yes No Maybe

Don't know N/A

1. Did your mentor help you identify tangible steps to meet your goals and objectives?
2. Did your mentor connect you to other professionals who could "fill in the gaps" in areas where you might be less skilled?

*Mark only one oval.*

Yes No Maybe

Don't know N/A

1. Did your mentor stay engaged and invested in your development and in the relationship?

*Mark only one oval.*

Yes No Maybe

Don't know N/A

1. Did your mentor exhibit integrity?
2. Did your mentor hold each of you to high standards?

*Mark only one oval.*

Yes No Maybe

Don't know N/A

1. Did you establish a written agreement including goals to be met under the direction or guidance of your mentor?

*Mark only one oval.*

Yes No Maybe

Don't know N/A

1. Were you satisfied with the mentor we/you selected and matched?

*Mark only one oval.*

Yes No Maybe

Don't know N/A

1. Did the two of you determine at the beginning of the relationship, guidelines by which to evaluate the success of the relationship?

*Mark only one oval.*

Yes No Maybe

Don't know N/A

1. Did you and your mentor complete the goals planned?

*Mark only one oval.*

Yes No Maybe

Don't know N/A

1. Were you happy with the frequency of meetings?

*Mark only one oval.*

Yes No Maybe

Don't know N/A

1. Were you happy with the style of mentoring in your relationship?

*Mark only one oval.*

Yes No Maybe

Don't know N/A

1. Did the relationship meet your expectations?

*Mark only one oval.*

Yes No Maybe

Don't know N/A

Directions: Describe in the box using your own words, what ever length you may need to express your answers.

PART 2: Your personal statements about your mentor

1. Your Partnership

1a. What were/are two of the most beneficial development activities you did/do?

1b. What is the most beneficial change you identified in yourself as a result of your mentorship?

1. Personal Growth

2a. As the result of having a mentor, I’ve gained the following knowledge, skills, and/or attitude change:

2b. Other benefits I’ve received from this mentoring relationship:

2c. Something I plan to do or have done more of as the result of the relationship:

1. Our relationship

3a. Ways, if any, this mentoring partnership could be more effective:

3b. Recommendations I’d make to other mentor-mentee pairs:

3c. General Comments on the mentoring initiative or partnership:
