# Supplementary material for: A national program to advance dementia research in Vietnam
Source: BMC Health Serv Res. 2024 Feb 1;24:156. doi: 10.1186/s12913-024-10608-w (PMC10832103; doi:10.1186/s12913-024-10608-w)
Supplement: Supplementary file 3 — Supplementary Material 3 [file 12913_2024_10608_MOESM3_ESM.docx]

VAN MENTORING PROGRAM

Dear Mentors,

Thank you so much for participating as one of our faculty mentors for our NIH R01 grant project "Advancing Alzheimer’s Family Caregiving Interventions and Research Capacity in Vietnam"!

Please take a few moments to complete the survey questions below. Your responses will remain completely anonymous. Your feedback will help us improve our mentoring program to support Vietnamese investigators who conduct dementia research in Vietnam.

If you have any questions, please contact us at [vanprograms@gmail.com](mailto:vanprograms@gmail.com). Sincerely,

Vietnam Alzheimer’s and other dementias research Network (VAN) Steering Committee

Part 1: Survey

Directions: Click one selection per question which best describes your opinion regarding your mentee.

1. Was your mentee easy to approach and talk with?

*Mark only one oval.*

Yes No Maybe

Don't know N/A

1. Did the two of you discuss your mentee’s career/individual development plan or just focus on undertaking their funded pilot project?

*Mark only one oval.*

Career/individual development plan

Focus on undertaking the funded pilot project Both

1. Did your mentee consider your advice and accept encouragement from you with respect to his/her goals and objectives?

*Mark only one oval.*

Yes No Maybe

Don't know N/A

1. Did the two of you meet regularly?

*Mark only one oval.*

Yes No Maybe

Don't know N/A

5a. If answer is Yes to question 4: How often did the two of you meet?

5b. If answer is No to question 4: Why was that?

1. Did you ﬁnd the meetings productive?

*Mark only one oval.*

Yes No Maybe

Don't know N/A

1. Did your mentee send you an agenda prior to meetings?

*Mark only one oval.*

Yes No Maybe

Don't know N/A

1. Did you solicit your mentee's thoughts and opinions when making suggestions or recommendations?

*Mark only one oval.*

Yes No Maybe

Don't know N/A

1. Did you help your mentee identify tangible steps to meet their goals and objectives?

*Mark only one oval.*

Yes No Maybe

Don't know N/A

1. Did you connect your mentee with other professionals who could "ﬁll in the gaps" in areas where they might be less skilled?

*Mark only one oval.*

Yes No Maybe

Don't know N/A

1. Did your mentee stay engaged and invested in meeting the relationship objectives?

*Mark only one oval.*

Yes No Maybe

Don't know N/A

1. Did your mentee respect your time and relationship boundaries (e.g., privacy, frequency of communication)?

*Mark only one oval.*

Yes No Maybe

Don't know N/A

1. Did you feel mentoring was a worthwhile endeavor?

*Mark only one oval.*

Yes No Maybe

Don't know N/A

1. Were you satisﬁed with the mentoring relationship?

*Mark only one oval.*

Yes No Maybe

Don't know N/A

1. Did the two of you determine at the beginning of the relationship, guidelines by which to evaluate the success of the relationship?

*Mark only one oval.*

Yes No Maybe

Don't know N/A

1. Did you and your mentee complete the goals planned?

*Mark only one oval.*

Yes No Maybe

Don't know N/A

1. Were you happy with the frequency of meetings?

*Mark only one oval.*

Yes No Maybe

Don't know N/A

1. Were you happy with the style of mentoring in your relationship?

*Mark only one oval.*

Yes No Maybe

Don't know N/A

1. Did the relationship meet your expectations?

*Mark only one oval.*

Yes No Maybe

Don't know N/A

Part 2: Your personal statements about your mentee

Directions: Describe in the box using your own words, what ever length you may need to express your answers.

1. Your Partnership

1a. What were/are two of the most beneficial development activities you did/do?

1b. What is the most beneﬁcial change you identiﬁed in yourself as a result of your mentorship?

1. Personal Growth

2a. As the result of having a mentee, I’ve gained the following knowledge, skills, and/or attitude change:

2b. Other beneﬁts I’ve received from this mentoring relationship:

2c. Something I plan to do or have done more of as the result of the relationship:

1. Our relationship

3a. Ways, if any, this mentoring partnership could be more effective:

3b. Recommendations I’d make to other mentor-mentee pairs:

3c. General Comments on the mentoring initiative or partnership:
