# Supplementary material for: A national program to advance dementia research in Vietnam
Source: BMC Health Serv Res. 2024 Feb 1;24:156. doi: 10.1186/s12913-024-10608-w (PMC10832103; doi:10.1186/s12913-024-10608-w)
Supplement: Supplementary file 4 — Supplementary Material 4 [file 12913_2024_10608_MOESM4_ESM.docx]

Final Progress Report

# Section A: Cover Page

| A.1: PROJECT DETAILS | | | | |
| --- | --- | --- | --- | --- |
| Project Title | |  | | |
| Principal Investigator Information | | Name:  Email:  Phone number: | | |
| Recipient Organization Information | | Organization name:  Address: | | |
| Approved year | |  | | |
| Project Period  (according to the contract) | | Start: MM/DD/YYYY  End: MM/DD/YYYY | | |
| Approved budget | | USD:  VND: | | |
| Project’s research team | |  | | |
| A.2: PROJECT STATUS – Please select your reason for completing this form | | | | |
| *Status* | ***Select*** | | ***Indicate Date*** | ***Note*** |
| Completed |  | | Date of completion: |  |
| Extension required |  | | Anticipated Date of completion: |  |
| Not commenced |  | | Date work was stopped: |  |
| In progress |  | | Anticipated Date of completion: |  |

# Section B: Accomplishments

| B.1. What are the major goals of the project?  *List the major goals of the project as stated in the approved application or as approved by the VAN Steering committee* |  |
| --- | --- |
| Have the major goals changed since the initial competing award or previous report? | YES  NO |
| If YES, please provide a revised description of major goals/specific aims |  |
| B.2 What was accomplished under these goals? | *1) major activities:* |
|  | *2) specific objectives:* |
|  | *3) significant results, including major findings, developments, or conclusions (both positive and negative):* |
|  | *4) key outcomes or other achievements:* |
| B.3 Competitive Revisions/Administrative Supplements.  *Is there one or more Revision/Supplement associated with this award for which reporting is required?* | YES  NO |
| If YES, identify the Revision(s)/ Supplements(s) by grant number or title and describe the specific aims and accomplishments for each Revision/ Supplement funded during this reporting period. |  |
| B.4 What opportunities for training and professional development has the project provided?  *If the research is not intended to provide training and professional development opportunities or there is nothing significant to report during the reporting period, select NOTHING TO REPORT* | YES  NOTHING TO REPORT |
| If YES, describe opportunities for training and professional development provided to anyone who worked on the project or anyone who was involved in the activities supported by the project. |  |
| B.5 How have results been disseminated to communities of interest? | YES  NOTHING TO REPORT |
| If YES, describe how the results have been disseminated to communities of interest. Include any outreach activities. |  |

# Section C: Products

| C.1 Publications.  *Are there publications or manuscripts accepted for publication in a journal or other publication (e.g., book, one-time publication, monograph, preprint) resulting directly from the award?* | YES  NO |
| --- | --- |
| If YES, please provide the full citation(s). |  |
| C.2 Website(s) or other internet site(s).  *For awards not designed to create or maintain one or more websites, select NOTHING TO REPORT* | YES  NOTHING TO REPORT |
| If YES, list the URL for any Internet site(s) that disseminates the results of the research activities. A short description of each site should be provided.  It is not necessary to include the publications already provided in C.1. |  |
| C.3 Technologies or techniques. | YES  NOTHING TO REPORT |
| If YES, identify technologies or techniques that have resulted from the research activities. Describe the technologies or techniques and how they are being shared*.* |  |
| C.4 Inventions, patent applications and/or licenses.  *Have inventions, patent applications and/or licenses resulted from the award during this reporting period?* | YES  NO |
| If YES, has this information been previously provided to the PHS or to the official responsible for patent matters at the grantee organization? | YES  NO |
| C.5 Other products and resources.  (Such as submitted papers to journal, presentation submitted and accepted…) | YES  NOTHING TO REPORT |
| If YES, identify any other significant products that were developed under this project, such as audio or video, data or databases, software, instruments or equipment, etc. |  |

# Section D: Impact

*Impact will be used to describe ways in which the work, findings, and specific products of the project have had an impact (on policy and practice).*

| D.1. What is the impact on physical, institutional, or information resources that form infrastructure?  *Describe ways, if any, in which the project made an impact, or is likely to make an impact, on…* | - Physical resources (such as facilities, laboratories, or instruments): - Institutional resources (such as establishment or sustenance of societies or organizations): - Information resources, electronic means for accessing such resources or for scientific communication: - Policy (institution or wider context): - Practice: - Other: |
| --- | --- |

# Section E: Changes

| E.1. Actual or anticipated challenges or delays and actions or plans to resolve them. | YES  NOTHING TO REPORT |
| --- | --- |
| If YES, describe challenges or delays encountered during the reporting period and actions or plans to resolve them. |  |
| E.2. Significant changes to human subjects, vertebrate animals, biohazards, and/or select agents. | YES  NOTHING TO REPORT |
| If YES, describe significant deviations, unexpected outcomes, or changes in approved protocols for human subjects, biohazards and/or select agents |  |

# Section F: Budget

**Reporting following the proposed budget justification**

# Section G: Outcomes

**What were the outcomes of the award?**

Outcomes will be made **publicly available**, thus allowing recipients to provide the general public with a concise summary of the cumulative outcomes or findings of the project at the end of a competitive segment. For NIH awards the length should **not** exceed half a page. In addition, for the interim or final RPPR the summary of outcomes or findings of the award must be written in the following format:

- Is written for the general public in clear, concise, and comprehensible language;
- Is suitable for dissemination to the general public, as the information may be available electronically;
- Does not include proprietary, confidential information or trade secrets

Please refer to the following link for samples of acceptable [project outcomes](https://grants.nih.gov/grants/rppr/sample_project_outcomes_RPPR.htm).
