# Supplementary material for: A national program to advance dementia research in Vietnam
Source: BMC Health Serv Res. 2024 Feb 1;24:156. doi: 10.1186/s12913-024-10608-w (PMC10832103; doi:10.1186/s12913-024-10608-w)
Supplement: Supplementary file 5 — Supplementary Material 5 [file 12913_2024_10608_MOESM5_ESM.docx]

**List of funded projects (n=21)**

| **Alzheimer's Disease and Alzheimer's Disease Related Dementias (AD/ADRD) prevention and risk factors (n=5)** |
| --- |
| 1. Capacity of health system in response to prevention and control of dementia in older persons: Case studies in the North of Vietnam, 2020 |
| 1. Dementia and war's long-term impacts among elderly people in Northern Vietnam |
| 1. Association between 24h blood pressure variability and dementia in elderly hypertensive patients |
| 1. Cognitive impairment in the elderly after COVID-19 infection in Hanoi |
| 1. Cognitive impairment after critical illness in older patients |
| **AD/ADRD detection, screening, and diagnosis (n=4)** |
| 1. Non-invasive blood tests to identify RNA fingerprints of Alzheimer’s disease |
| 1. Normative data for common neuropsychological tests used in evaluating dementia from cognitively healthy Vietnamese |
| 1. Applying the CAIDE (Cardiovascular Risk Factors, Aging and Incidence of Dementia) scores in screening for people at risk of dementia in the community |
| 1. Validation of MoCA (Montreal Cognitive Assessment) in Vietnam |
| **Supports for people living with AD/ADRD (n=10)** |
| 1. A multidomain intervention program for older people with dementia |
| 1. Care needs assessment of older adults with dementia in Thach That District, Hanoi |
| 1. Efficacy of paper-based cognitive training in Vietnamese patients with early Alzheimer’s disease |
| 1. Assessing the nutritional status, eating disturbance issues, and proposing nutritional care interventions for patients with dementia in Vietnam |
| 1. Polypharmacy among persons with dementia in Vietnam: epidemiology, risk factors, and mortality |
| 1. Cost of treatment and care for dementia in Vietnam |
| 1. Comprehensive Geriatric Assessment (CGA) in older patients with cognitive impairment |
| 1. Preparedness of nursing students in taking care of dementia patients in Vietnam |
| 1. What is a good end-of-life with dementia? Perspectives of stakeholders in Vietnam |
| 1. Assessment of the stability and dissolution characteristics of donepezil tablets |
| **Supports for caregivers (n=2)** |
| 1. Smartphone app-based intervention for reducing stress, depression, and anxiety in caregivers of people with dementia in Vietnam |
| 1. Assessing family dementia caregivers' needs for social work services |
